# Imaging markers of small vessel disease and brain frailty, and outcomes in acute stroke

**DOI:** 10.1212/WNL.0000000000008881

**Published:** 2020-02-04

**Authors:** Jason P. Appleton, Lisa J. Woodhouse, Alessandro Adami, Jennifer L. Becker, Eivind Berge, Lesley A. Cala, Ana M. Casado, Valeria Caso, Hanne K. Christensen, Robert A. Dineen, John Gommans, Panos Koumellis, Szabolcs Szatmari, Nikola Sprigg, Philip M. Bath, Joanna M. Wardlaw, for the ENOS Investigators

**Affiliations:** From the Stroke Trials Unit (J.P.A., L.J.W., N.S., P.M.B.) and Radiological Sciences Research Group (R.A.D.), Division of Clinical Neurosciences, University of Nottingham; Stroke (J.P.A., N.S., P.M.B.), Nottingham University Hospitals NHS Trust, UK; Stroke Center (A.A.), IRCSS Sacro Cuore–Don Calabria Hospital, Negrar, Verona, Italy; Department of Medical Imaging (J.L.B.), College of Medicine, University of Arizona, Tucson; Department of Internal Medicine and Cardiology (E.B.), Oslo University Hospital, Norway; School of Medicine (L.A.C.), University of Western Australia, Crawley; Department of Neuroradiology (A.M.C.), Division of Clinical Neurosciences, Western General Hospital, Edinburgh, UK; Stroke Unit (V.C.), Santa Maria della Misericordia Hospital, University of Perugia, Italy; Neurology (H.K.C.), Bispebjerg and Frederiksberg Hospital, Copenhagen, Denmark; Department of Medicine (J.G.), Hawke's Bay District Health Board, Hastings, New Zealand; Department of Neuroradiology (P.K.), Nottingham University Hospitals, Queen's Medical Centre, UK; Department of Neurology (S.S.), Clinical County Emergency Hospital, Targu Mures, Romania; and Division of Neuroimaging Sciences (J.M.W.), Centre for Clinical Brain Sciences, Dementia Research Institute, University of Edinburgh, UK.

## Abstract

**Objective:**

To assess the association of baseline imaging markers of cerebral small vessel disease (SVD) and brain frailty with clinical outcome after acute stroke in the Efficacy of Nitric Oxide in Stroke (ENOS) trial.

**Methods:**

ENOS randomized 4,011 patients with acute stroke (<48 hours of onset) to transdermal glyceryl trinitrate (GTN) or no GTN for 7 days. The primary outcome was functional outcome (modified Rankin Scale [mRS] score) at day 90. Cognition was assessed via telephone at day 90. Stroke syndrome was classified with the Oxfordshire Community Stroke Project classification. Brain imaging was adjudicated masked to clinical information and treatment and assessed SVD (leukoaraiosis, old lacunar infarcts/lacunes, atrophy) and brain frailty (leukoaraiosis, atrophy, old vascular lesions/infarcts). Analyses used ordinal logistic regression adjusted for prognostic variables.

**Results:**

In all participants and those with lacunar syndrome (LACS; 1,397, 34.8%), baseline CT imaging features of SVD and brain frailty were common and independently associated with unfavorable shifts in mRS score at day 90 (all participants: SVD score odds ratio [OR] 1.15, 95% confidence interval [CI] 1.07–1.24; brain frailty score OR 1.25, 95% CI 1.17–1.34; those with LACS: SVD score OR 1.30, 95% CI 1.15–1.47, brain frailty score OR 1.28, 95% CI 1.14–1.44). Brain frailty was associated with worse cognitive scores at 90 days in all participants and in those with LACS.

**Conclusions:**

Baseline imaging features of SVD and brain frailty were common in lacunar stroke and all stroke, predicted worse prognosis after all acute stroke with a stronger effect in lacunar stroke, and may aid future clinical decision-making.

**Identifier:**

ISRCTN99414122.

Cerebral small vessel disease (SVD) is a common cause of lacunar ischemic stroke, hemorrhagic stroke, vascular cognitive impairment, and dementia.^1^ The pathophysiology of SVD differs from that of other stroke subtypes and is thought to reflect intrinsic damage to small perforating arterioles manifested as endothelial dysfunction, blood-brain barrier breakdown, and inflammation.^2^ Imaging markers of SVD include white matter hyperintensities or leukoaraiosis, microbleeds, prominent perivascular spaces, and lacunes, in addition to acute lacunar infarcts and intracerebral hemorrhage.^3^ All are visible on MRI, while microbleeds and perivascular spaces are not visible on CT scanning.

Recently, instead of considering each individual SVD feature separately, a summary score of SVD features was associated with risk factors,^4^ cognition,^5^ and mobility.^6^ Several large trials have reviewed the association between imaging markers of SVD and outcome.^7–9^ Some analyses focused on features seen on MRI,^4,9^ while others identified general prestroke features visible on CT (including SVD specific) that were associated independently with poor outcome (leukoaraiosis, cerebral atrophy, and old vascular lesions/infarcts), suggesting that these might represent markers of brain frailty.^7^

Few trials or large observational studies have focused on outcomes after acute lacunar stroke, largely because such patients have better clinical outcomes compared to patients with other ischemic stroke subtypes.^10–12^ Three trials have reported outcomes after acute lacunar stroke in a total of 835 participants,^10,12^ with 422 being the largest trial population of patients with acute lacunar stroke to date.^12^ There are also modest studies using nonrandomized data from thrombolysis registers.^10,11^ Although the Secondary Prevention of Small Subcortical Strokes (SPS3) trial recruited 3,020 patients up to 180 days after symptomatic lacunar stroke, it does not provide data on outcome after acute lacunar stroke.^13^ In contrast, the Efficacy of Nitric Oxide in Stroke (ENOS) trial assessed the safety and efficacy of transdermal glyceryl trinitrate (GTN) in 4,011 patients with acute stroke, of which a large proportion (1,397, 35%) were of the lacunar subtype.^14^

The aims of the present analysis were to assess the influence of imaging markers of SVD and brain frailty at baseline on functional and cognitive outcome in patients with acute stroke, particularly lacunar stroke, in the ENOS trial. We sought to test the following hypotheses: (1) baseline imaging markers of SVD will be more prevalent in those with lacunar than nonlacunar stroke; (2) increased baseline SVD score will be associated with poor functional and cognitive outcomes after acute lacunar stroke; and (3) increased baseline brain frailty score will be associated with poor functional and cognitive outcomes after acute lacunar and nonlacunar stroke.

## Methods

Details on the ENOS trial protocol, statistical analysis plan, baseline characteristics, and main results have been published previously.^14–17^ In summary, ENOS recruited 4,011 patients in 173 centers in 23 countries within 48 hours of stroke onset with high systolic blood pressure (140–220 mm Hg) and randomized them to GTN 5 mg patch or no patch for 7 days. Participants on antihypertensive medication before their index event were also randomized to continue or stop these medications for 7 days.

### Standard protocol, approvals, registrations, and patient consents

Patients or relatives/caregivers provided written consent. ENOS was registered (ISRCTN99414122) and approved by ethics committees/competent authorities in all participating countries.

### Study population

Stroke syndrome was assessed at baseline with the Oxfordshire Community Stroke Project (OCSP) clinical classification.^18^ We incorporated imaging findings to create a hierarchy of increasing specificity of definitions of acute lacunar stroke^19^ as follows: patients without lacunar syndrome (LACS; n = 2,614); patients with clinical LACS (n = 1397), including ischemic and hemorrhagic stroke; patients with LACS with a compatible scan (n = 623), that is, LACS with an adjudicated acute lacunar infarct or, if no acute infarct visible, then no alternative pathology seen to explain the presentation; and patients with LACS with corresponding acute lacunar infarction on imaging (n = 143). Therefore, LACS with corresponding acute lacunar infarction is a subset of LACS with compatible scan, and both are subsets of LACS.

### Imaging

CT or MRI brain scans were performed at baseline, usually before randomization. A second CT brain scan was performed at day 7 (end of treatment) when feasible. Scans were sent to the coordinating center and adjudicated with a set proforma^7^ by trained neuroradiologists or neurologists (A.A., J.L.B., L.A.C., A.C., R.A.D., P.K., J.M.W.) blinded to clinical details and randomization allocation. These assessments documented the location, size, and swelling associated with any acute ischemic or hemorrhagic lesion and the presence of prestroke changes, including atrophy, leukoaraiosis, and old vascular lesions. Atrophy was assessed separately in cortical and central regions, defined as 0 = absent, 1 = moderate, or 2 = severe, and compared against a standard template,^7^ thus providing a maximum score of 4. Leukoaraiosis was assessed separately in anterior and posterior brain regions,^20^ defined as 0 = no lucency, 1 = lucency restricted to region adjoining ventricles, or 2 = lucency covering entire region from lateral ventricle to cortex, providing a maximum score of 4. Old vascular lesions/infarcts were classified by location (e.g., cortical, striatocapsular, border zone, lacunar).

In addition to individual imaging markers of SVD, we applied scores adapted for CT scanning as follows: SVD score comprises 1 point each for severe leukoaraiosis (score = 2 anteriorly and/or posteriorly as above), severe atrophy (score = 2 cortically and/or centrally), and any old lacunar infarcts/lacunes (maximum 3 of 3).^9^ SVD score excluding atrophy (maximum 2 of 2) was also assessed because atrophy, although related, is not specific to SVD. Brain frailty score comprises 1 point each for leukoaraiosis (score = 1 or 2 anteriorly and/or posteriorly), cerebral atrophy (score = 1 or 2 cortically and/or centrally), and old vascular lesions/infarcts (maximum 3 of 3). Although there is no accepted definition of brain frailty, we used the individual features on both CT and MRI included in the Third International Stroke Trial, which were individually shown to be associated with poor clinical outcome after acute stroke.^7^

### Clinical outcomes

The primary outcome was the modified Rankin Scale (mRS) score,^21^ a 7-level ordered categorical scale (0 = independent, 6 = dead) measured at day 90. Secondary outcomes at day 90 included disability (Barthel Index), quality of life (health utility status calculated with European Quality of Life 5-dimensions 3-level scale and visual analog scale), mood (Zung Depression Scale), and cognition (telephone Mini-Mental State Examination [t-MMSE],^22^ modified Telephone Interview for Cognitive Status [TICS-M],^23^ and verbal fluency [animal naming]). In essence, t-MMSE and TICS-M assess attention and memory, while verbal fluency assesses executive function. Participants who died by day 90 were assigned the worst score for these outcomes.^24^ Safety outcomes included all-cause mortality, early neurologic deterioration (reduction of ≥5 points or reduction in the consciousness domain of >2 points from baseline to day 7 on the Scandinavian Stroke Scale [SSS]), and symptomatic hypotension. Day 90 outcomes were assessed by trained blinded assessors via telephone at national coordinating centers.

### Statistical analysis

Data were analyzed by intention to treat^16^ and are given as number (percent), median (interquartile range), or mean (SD). Differences in baseline characteristics were assessed with the χ^2^ test for categorical variables and 1-way analysis of variance for continuous variables.

Differences between treatment group (GTN vs no GTN) effects on outcome were assessed with binary logistic regression, multiple linear regression, ordinal logistic regression, or Cox proportional hazard regression. Associations between baseline imaging characteristics and mRS scores and cognitive outcomes at day 90 were assessed with ordinal logistic regression and multiple linear regression, respectively. Statistical models were adjusted for baseline prognostic covariates, including age, sex, baseline mRS score, history of stroke, history of diabetes mellitus, final diagnosis, nitrate use, baseline systolic blood pressure, baseline SSS score, thrombolysis, feeding status, time to randomization, and treatment allocation (GTN vs no GTN and/or continue vs stop prestroke antihypertensives). Results are reported as odds ratio (OR) or mean difference (MD) and associated 95% confidence intervals (CIs) or standardized regression coefficient (β) with significance defined as *p* ≤ 0.05. Analyses were performed with SPSS version 24 (SPS Inc, Chicago, IL).

### Data availability

The data that support the findings of this study are available from the corresponding author on reasonable request. Supplementary data are available from Dryad (tables 6–8 and figures 3–5, doi.org/10.5061/dryad.6552r2b).

## Results

A total of 1,397 of 4,011 (34.8%) patients with LACS were recruited into ENOS. Baseline characteristics of LACS differed from those presenting with non-LACS (table 1): compared to participants without LACS, those with LACS were younger (67.9 [12.0] years); more were male (61.1%), recruited in Asia, had diabetes mellitus, or were smokers; and they were less dependent at baseline, with less prestroke hypertension, TIA, hyperlipidemia, ischemic heart disease, peripheral arterial disease, or atrial fibrillation. Participants with LACS also had milder index events (mean SSS score 42 vs 29, *p* < 0.001) and a longer time to randomization, and fewer received thrombolysis than participants without LACS. Most participants (92%) were imaged with CT. At baseline, there were more acute lacunar infarctions and fewer acute hemorrhages in those with LACS than in those with non-LACS, while background changes—leukoaraiosis, cerebral atrophy and old vascular lesions—did not differ between the groups (data available from Dryad, table 6, doi.org/10.5061/dryad.6552r2b). In participants with LACS, 50% had moderate or severe cerebral atrophy, 41% had some degree of leukoaraiosis, and 61% had an old vascular lesion. SVD and brain frailty scores were moderately positively correlated (Spearman correlation coefficient 0.626, *p* < 0.001).

**Table 1:**
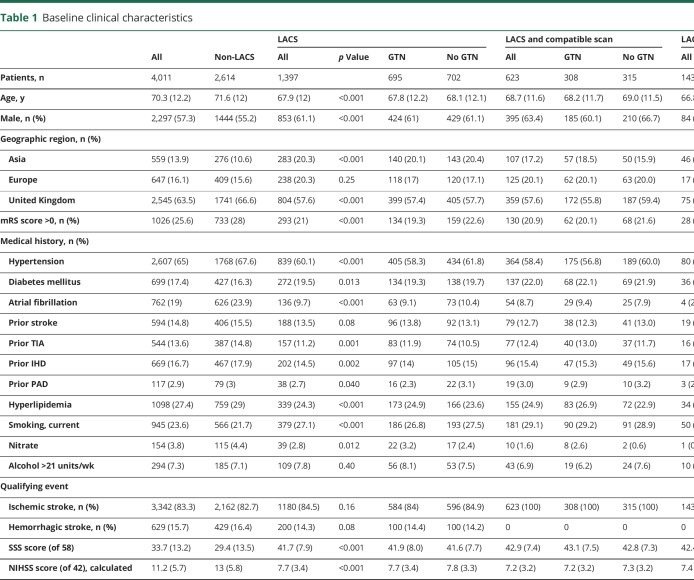

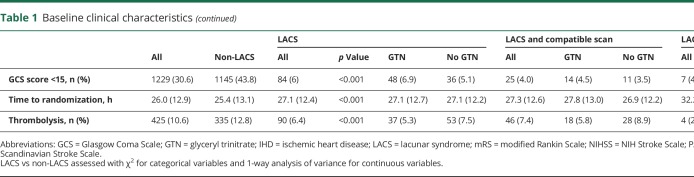
Baseline clinical characteristics

### Imaging and functional outcome

The associations between baseline imaging characteristics and mRS score at day 90 were assessed in all participants (n = 3,995), those with LACS (n = 1,392), those with LACS and a compatible scan (n = 623), and those with LACS with an acute lacunar infarction (n = 143) (table 2). In the whole population, visible infarction, cerebral atrophy score, leukoaraiosis score, old lacunar infarct/lacune, and old striatocapsular infarct were individually associated with unfavorable shifts in mRS score at day 90. In participants with LACS, parenchymal hemorrhage, cerebral atrophy score, leukoaraiosis score, old lacunar infarct/lacune, and old striatocapsular infarct were individually associated with unfavorable shifts in mRS score at day 90. In those with LACS and a compatible scan, cerebral atrophy, old lacunar infarct/lacune, and old striatocapsular infarct were individually associated with unfavorable shifts in mRS score at day 90. In those with LACS and acute lacunar infarction, no individual imaging features were associated with mRS score at day 90.

**Table 2:**
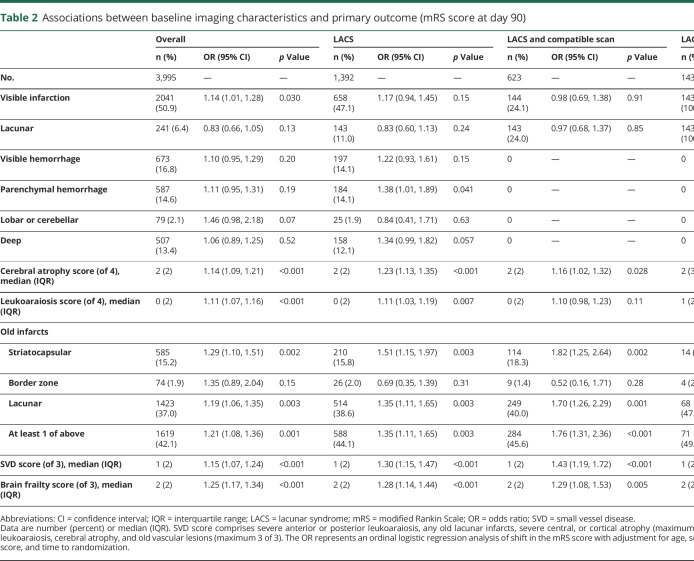
Associations between baseline imaging characteristics and primary outcome (mRS score at day 90)

Increasing SVD score was associated with unfavorable shifts in mRS score at day 90 in the whole ENOS population, those with LACS, and those with LACS and a compatible scan. Each 1-point increase in SVD score was associated with an increased odds of shift in the mRS score to more death or dependency, with increasing effect sizes present with increasing specificity of lacunar stroke diagnosis: whole ENOS population OR 1.15 (95% CI 1.07–1.24), LACS OR 1.30 (95% CI 1.15–1.47), LACS and a compatible scan OR 1.43 (95% CI 1.19–1.72), and LACS with acute lacunar infarction OR 1.45 (95% CI 1.00–2.11) (table 2). Removing atrophy from the SVD score resulted in comparable associations with outcome (data not shown). In a multivariate ordinal regression assessing the association between individual features comprising the SVD score and functional outcome in the whole ENOS population, all features had similar effect sizes within the model: severe leukoaraiosis OR 1.17 (95% CI 0.99–1.38), severe atrophy OR 1.12 (95% CI 0.99–1.27), and old lacunar infarcts/lacunes OR 1.17 (95% CI 1.04–1.32). However, with increasing specificity of lacunar stroke diagnosis, the effect sizes for severe atrophy and old lacunar infarcts/lacunes increased, while severe leukoaraiosis did not (severe atrophy: LACS OR 1.35 [95% CI 1.10–1.67], LACS and a compatible scan OR 1.39 [95% CI 1.02–1.89]; old lacunar infarcts/lacunes: LACS OR 1.31 [95% CI 1.07–1.61], LACS and a compatible scan OR 1.67 [95% CI 1.23–2.25]).

**Figure 1:**
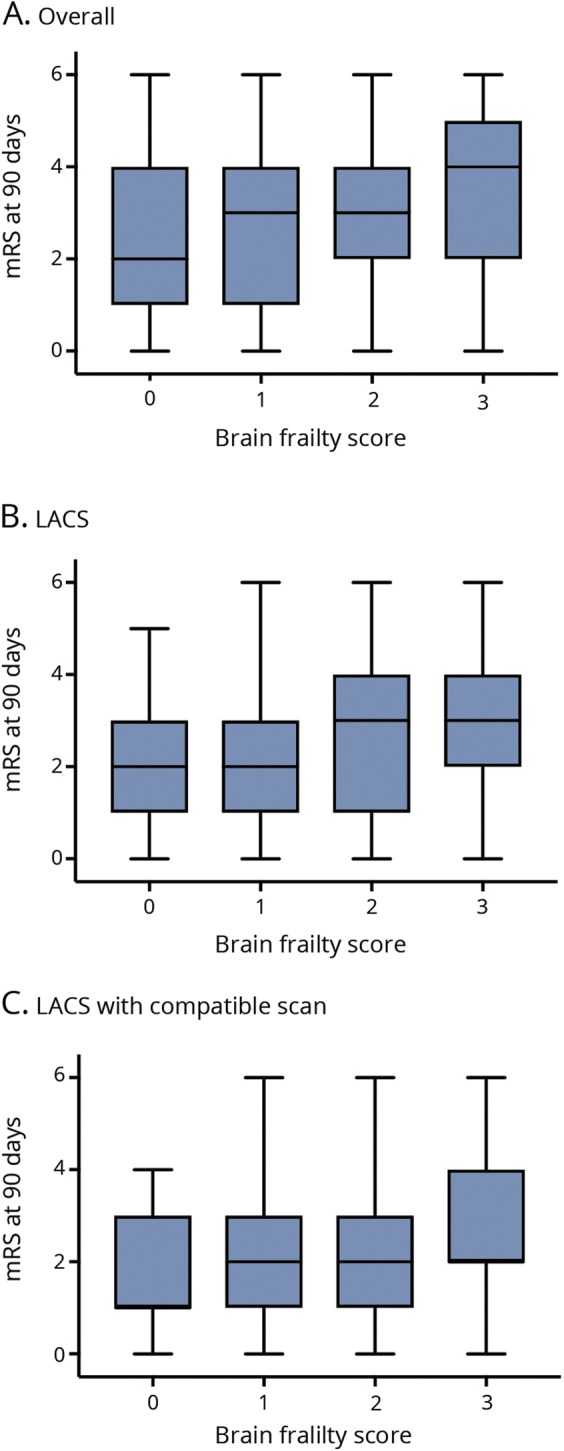
mRS score at day 90 by brain frailty score on baseline imaging Boxplots of modified Rankin Scale (mRS) score at day 90 by brain frailty score in (A) the whole Efficacy of Nitric Oxide in Stroke (ENOS) population (n = 3,995), (B) participants with lacunar syndrome (LACS, n = 1392), and (C) participants with LACS with compatible scan (n = 623).

Increased brain frailty features were associated with worse functional outcome at 90 days (figure 1) in the whole ENOS population (OR 1.25, 95% CI 1.17–1.34), in those with LACS (OR 1.28, 95% CI 1.14–1.44), and in those with LACS and a compatible scan (OR 1.29, 95% CI 1.08–1.53), but not in the small group of LACS with an acute lacunar infarct. Hence, a 1-point increase in brain frailty score was associated with a similar increased odds of shift to more death or dependency across the 3 populations. In a multivariate ordinal regression assessing the association between individual features comprising brain frailty and functional outcome in the whole ENOS population, all features had similar effect sizes within the model: leukoaraiosis OR 1.10 (95% CI 1.05–1.15), atrophy OR 1.12 (95% CI 1.06–1.19), and old vascular lesions/infarcts OR 1.14 (95% CI 1.01–1.29). Unlike with the SVD score, we did not see any consistent changes in the effect sizes for the individual features comprising the brain frailty score as we progressed through the lacunar stroke hierarchy.

MRI data were available for 109 participants with LACS. Only old lacunar infarcts/lacunes were associated with an unfavorable shift in mRS score at day 90 (data available from Dryad, table 7, doi.org/10.5061/dryad.6552r2b).

### Imaging and cognitive outcomes

Day 90 cognitive outcomes were available in about half of the overall population: t-MMSE in 1,949 (49%, table 3), TICS-M in 1,930 (48%, table 4), and verbal fluency in 2,269 (57%, table 5). Participants with no cognitive data at 90 days were older (73 [16] vs 71 [18] years) and had more severe strokes (baseline mean SSS score 35 vs 32, *p* < 0.001) than those with at least 1 telephone cognitive assessment.

**Table 3:**
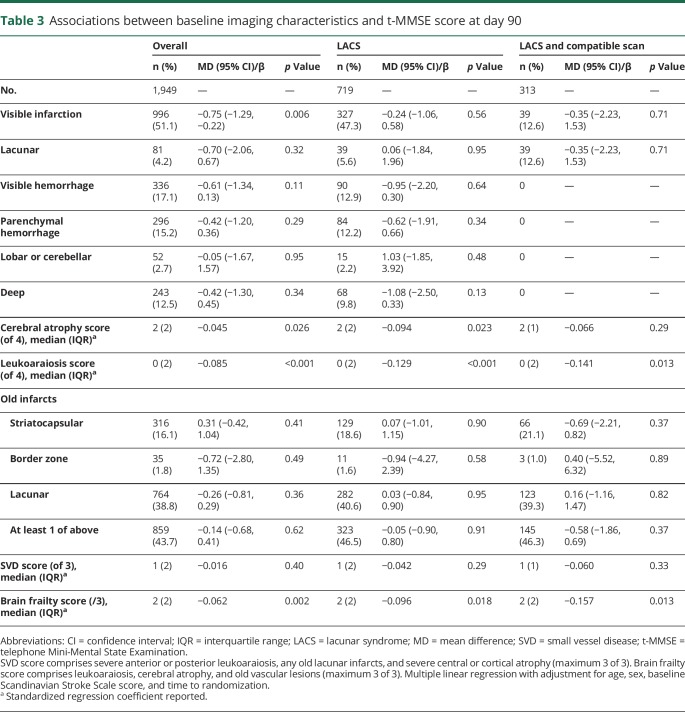
Associations between baseline imaging characteristics and t-MMSE score at day 90

**Table 4:**
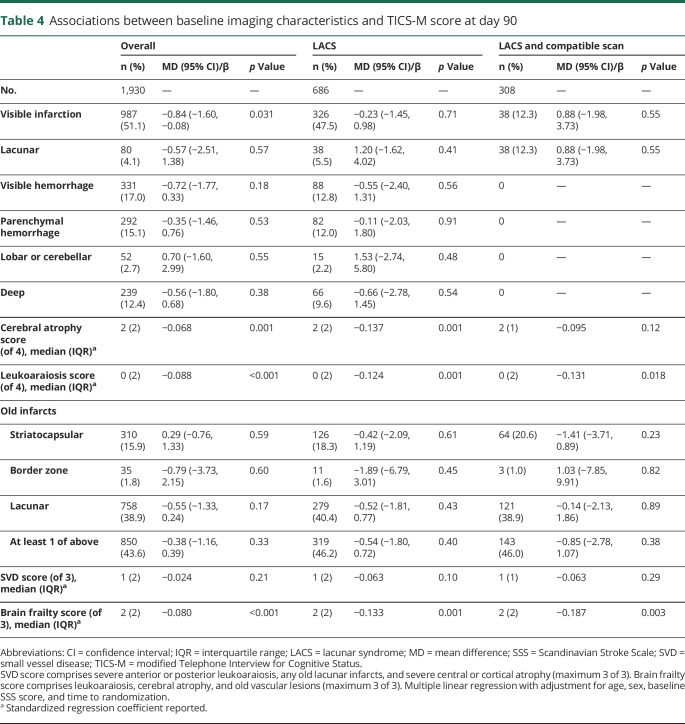
Associations between baseline imaging characteristics and TICS-M score at day 90

**Table 5:**
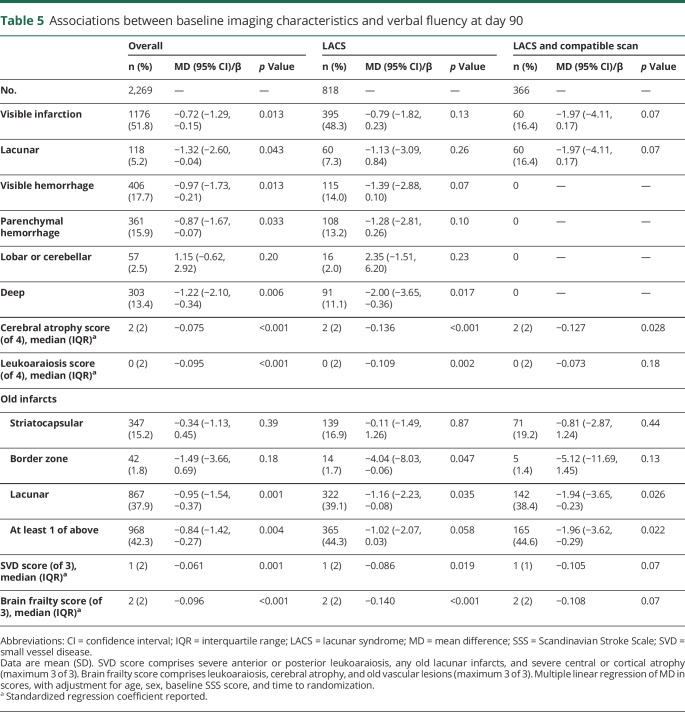
Associations between baseline imaging characteristics and verbal fluency at day 90

**Figure 2:**
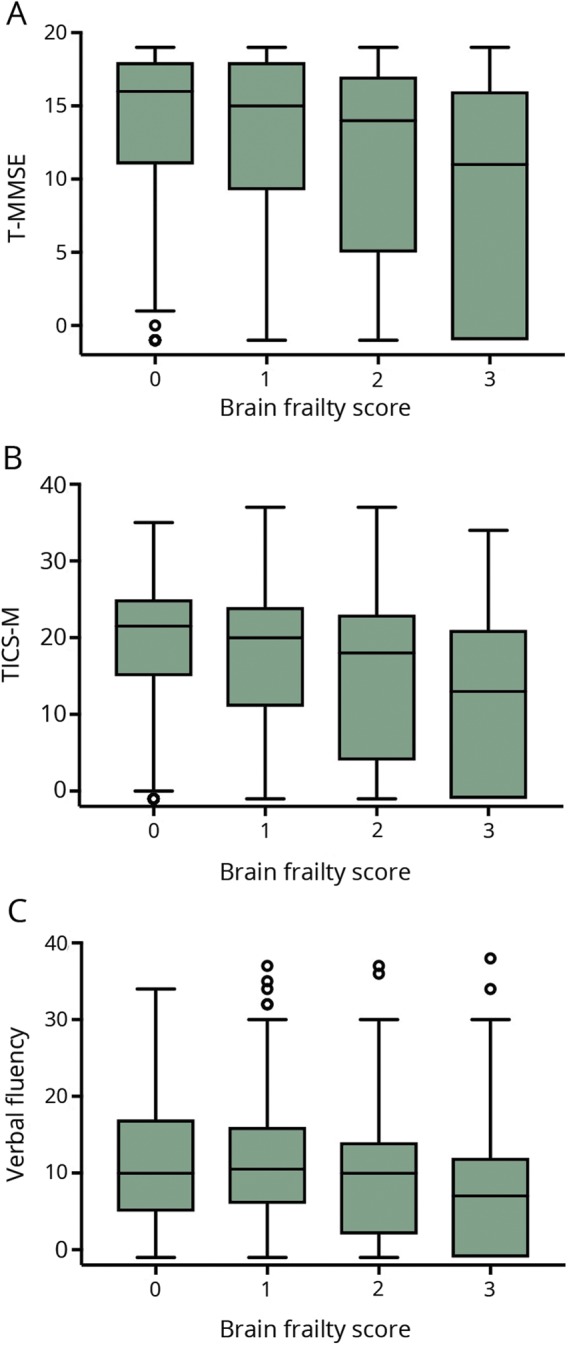
Cognitive scores at day 90 by brain frailty score on baseline imaging Boxplots of cognitive scores—(A) telephone Mini-Mental State Examination (t-MMSE, n = 1,949), (B) modified Telephone Interview for Cognitive Status (TICS-M, n = 1,930), and (C) verbal fluency (n = 2,269)—at day 90 by brain frailty in the whole Efficacy of Nitric Oxide in Stroke (ENOS) population. Lower cognitive scores indicate worse cognition.

Overall, visible infarction, cerebral atrophy score, and leukoaraiosis score were independently associated with worse cognitive scores on t-MMSE and TICS-M at 90 days (data available from Dryad, figures 3 and 4, doi.org/10.5061/dryad.6552r2b). In addition, acute lacunar infarction, parenchymal and deep hemorrhage, and old lacunar infarct/lacune were each associated independently with worse verbal fluency scores at 90 days. Brain frailty was associated with worse scores on all 3 cognitive measures (figure 2). Those with baseline brain frailty scores of 3 had worse cognitive scores compared to those with no evidence of brain frailty (t-MMSE: MD −1.13, 95% CI −2.37 to 0.11, *p* = 0.07; TICS-M: MD −2.49, 95% CI −4.25 to −0.73, *p* = 0.006; verbal fluency: MD −1.72, 95% CI −2.97 to −0.46, *p* = 0.008). In multivariate linear regression assessing the association between individual features comprising brain frailty and cognition, leukoaraiosis, and atrophy were consistently associated with worse cognition across all 3 measures, while old vascular lesions/infarcts were not. In contrast to brain frailty, SVD score was associated with worse verbal fluency only: those with an SVD score of 3 had lower verbal fluency scores compared to those with an SVD score of 0 (MD −2.12, 95% CI −3.66 to −0.58, *p* = 0.007, data available from Dryad figure 5, doi.org/10.5061/dryad.6552r2b). In multivariate analyses assessing the association between individual features of the SVD score and cognitive outcomes, only severe leukoaraiosis was associated with all 3 cognitive measures, while old lacunar infarcts/lacunes were associated with worse verbal fluency only.

In participants with LACS, leukoaraiosis and cerebral atrophy scores were associated with worse cognitive scores on all 3 measures (tables 3–5). Brain frailty score was associated with worse cognitive outcomes across all 3 domains. Those with baseline brain frailty scores of 3 had worse cognitive scores compared to those with no brain frailty (t-MMSE: MD −1.92, 95% CI −3.89 to 0.06, *p* = 0.057; TICS-M: MD −4.26, 95% CI −7.17 to −1.36, *p* = 0.004; verbal fluency: MD −3.81, 95% CI −6.11 to −1.51, *p* = 0.001). As in the whole ENOS population, in multivariate linear regression models, the individual brain frailty markers leukoaraiosis and atrophy were consistently associated with worse cognition across all 3 measures, while old vascular lesions/infarcts were not. SVD score was associated with worse verbal fluency only (tables 3–5), which in multivariate analyses seemed to be driven by the effect of old lacunar infarcts/lacunes rather than severe leukoaraiosis or severe atrophy.

In those with LACS and a compatible scan, leukoaraiosis score was associated with worse t-MMSE and TICS-M scores, while cerebral atrophy score was associated with worse verbal fluency scores only. Brain frailty score was associated with worse t-MMSE and TICS-M scores but was not significantly associated with verbal fluency. SVD scores were not associated with cognitive outcomes in this small population (tables 3–5).

There were insufficient cognitive data for analysis in those with LACS and an acute lacunar infarction (n = 60).

### GTN and lacunar stroke

GTN had no effect on mRS score at day 90 compared with no GTN in those with LACS (OR 1.07, 95% CI 0.88–1.29, n = 1392), LACS and compatible scan (OR 1.09, 95% CI 0.82–1.45, n = 623), or LACS and acute lacunar infarction (OR 1.00, 95% CI 0.53–1.87, n = 143). GTN within 6 hours did not change any day 90 clinical outcomes in lacunar stroke populations (data available from Dryad, table 8, doi.org/10.5061/dryad.6552r2b). Furthermore, in 339 participants with LACS, GTN did not influence imaging markers at day 7 (data not shown).

## Discussion

In this large population of patients with lacunar stroke syndromes randomized into the ENOS trial, baseline imaging markers of SVD and brain frailty were common and associated with poor functional and cognitive outcomes at 90 days, individually and in combination. The magnitude of SVD and brain frailty was similar in those with lacunar and nonlacunar stroke despite the patients with lacunar stroke being younger. Furthermore, the strength of association of SVD score with poor functional outcome increased with increasing specificity of lacunar stroke diagnosis, while brain frailty had similar associations across the trial population. GTN did not alter functional outcome of patients with acute stroke presenting with LACS.

Prestroke TIA, ischemic heart disease, peripheral arterial disease, and atrial fibrillation were all less common in participants with lacunar stroke than in those with nonlacunar strokes, supporting previous studies demonstrating that large artery disease and cardioembolic sources are important risk factors for nonlacunar, but less so for lacunar ischemic strokes.^19,25,26^ While smoking and diabetes mellitus were more common in our lacunar than nonlacunar stroke population,^4^ perhaps surprisingly, those with LACS had less hypertension and hyperlipidemia than those with non-LACS, although this finding is in keeping with previous data showing that traditional vascular risk factors, when combined, account for <2% of the variance in SVD features in patients with stroke and healthy older populations.^26^

In line with our results, SVD scores and their component imaging findings have been associated with adverse clinical outcomes after stroke in 4 other smaller cohorts. Data from the Stroke Imaging Repository (STIR)/Virtual International Stroke Trials Archive (VISTA) showed that severe leukoaraiosis and total SVD score on MRI in 259 patients with ischemic stroke treated with thrombolysis were associated with increased disability and functional dependency at 90 days.^9^ In contrast, lacunes, cerebral atrophy, and enlarged perivascular spaces were not individually associated with clinical outcome, probably because of a lack of power. A retrospective cohort involving 1,026 participants using MRI data found an association between SVD score and all-cause and stroke-related mortality.^27^ SVD burden on MRI has also been associated with worse quality-of-life scores 3 months after acute ischemic stroke^28^ and decreased cognitive function over 4 years in patients with first-ever lacunar ischemic stroke and hypertension.^29^ In the present analysis, SVD scores with and without atrophy showed similar associations with functional outcome at 90 days overall and in the lacunar stroke populations, suggesting that the main drivers for functional outcome were the vascular rather than the associated neurodegenerative imaging features. The strength of association of SVD score with worse functional outcome increased with increasing specificity of lacunar stroke diagnosis.

We confirm the important prognostic value of the 3 brain frailty measures on CT of leukoaraiosis, atrophy, and old vascular lesions that were each independently associated with poor outcome in IST-3.^7^ Furthermore, we have demonstrated that pooling these imaging markers in a score including any old infarct (not just lacunes as in the SVD score) was associated with functional and cognitive outcomes 90 days after stroke; in contrast to the SVD score, brain frailty showed a similar strength of association with poor functional outcome across non-LACS and LACS populations. Although SVD and brain frailty scores were moderately positively correlated and measure similar imaging markers, the differences in severity of the individual markers included provide 2 different ways of assessing brain health. This is supported by their differing effects on clinical outcomes and the different contribution of the individual features in multivariate regression models.

There were interesting differences in associations between imaging features and performance in the different cognitive domains. In the whole ENOS population, several acute and prestroke imaging features were associated with impairment in all cognitive domains. However, the SVD score added little compared with leukoaraiosis alone, which tended to be associated with verbal fluency, whereas brain frailty score was associated with all cognitive impairments. This may reflect the known effects of white matter lesions on processing speed and of brain atrophy (as a sign of neurodegeneration) on memory; alternatively, it could reflect a lack of statistical power due to missing cognitive data. Furthermore, these findings suggest that imaging signs may have differential relationships with cognition and its domains in different stroke subtypes; this emphasizes the importance of testing all cognitive domains in future research and that some cognitive tests may be insensitive to the types of impairment specific to particular stroke types.

These imaging markers are easy to detect on plain CT by physicians and radiologists in acute stroke and, given their strong prognostic significance, may prove useful for predicting outcome when added to clinical markers in clinical practice. Whether brain frailty on imaging correlates with clinical frailty is unclear, although these features correlate with gait, balance,^6^ and cognitive impairments,^5^ implying that a correlation with clinical frailty is likely; thus, these brain features may prove to be useful surrogate markers. For future acute stroke clinical trials, minimization based on baseline imaging markers of brain frailty could be important to balance these prognostic variables between treatment groups.

The favorable effect of GTN given within 6 hours on stroke onset^30,31^ was not seen in this analysis of participants with LACS. The Rapid Intervention With Glyceryl Trinitrate in Hypertensive Stroke Trial–2 (RIGHT-2, ISRCTN26986053) will provide further detail on whether the effects of GTN vary between differing stroke etiologies, with imaging markers being key secondary outcomes.^32,33^ In addition, the longer-term administration of isosorbide mononitrate (a long-acting nitrate) is being assessed for safety and efficacy in patients with lacunar ischemic stroke and SVD in the ongoing Lacunar Intervention Trial-2 (LACI-2, ISRCTN14911850).

The strengths of this ENOS analysis include the largest dataset of patients with acute lacunar stroke to date from a high-fidelity randomized controlled trial with near-complete follow-up, blinded and standardized adjudication of imaging by trained observers using a standardized proforma, ordinal analysis of the mRS score to increase statistical power, and generalizability to clinical practice through the predominant use of CT imaging. However, there are important limitations. First, no adjustment was made for multiplicity of testing. Therefore, some of the results may, in part, be due to chance, although the strength of associations seen mitigates the risks of multiple testing. Second, the mean age in ENOS was lower than that seen in many unselected clinical stroke populations, although this is typical for lacunar stroke. This may have attenuated the observed associations because brain frailty is likely to be even more prevalent in an older population. Third, ENOS recruited over a 12-year period in which clinical practice changed. Thus, the time from baseline imaging to stroke onset was longer than we would expect in current stroke clinical practice but is still common in patients with minor stroke. Fourth, MRI, which is more sensitive to features of SVD and acute infarction, was performed in only a small proportion of ENOS participants. However, the predominant use of CT enabled associations between SVD features visible on CT and outcome to be assessed, which were found to be in keeping with MRI-based studies and immediately applicable in clinical practice. Fifth, clinical stroke syndrome classification with the OCSP was investigator reported and not adjudicated centrally. In addition, the OCSP is known to misclassify ≈15% of lacunar strokes as partial anterior circulation syndrome and cortical strokes as LACS, thus adding noise to the data. We accounted for this in our more specific lacunar stroke populations; this added to the generalizability of the dataset and its findings to clinical practice. Sixth, telephone cognition data were available for about half of participants, largely because of stroke severity,^24^ limiting generalizability. Finally, although trained neuroradiologists adjudicated the imaging data, we cannot exclude interrater and intrarater variability over the time scale of the trial.

We add to the increasing body of evidence that baseline imaging markers of SVD and brain frailty are common and associated with worse functional and cognitive outcomes at 90 days individually and when amalgamated as scores. Whether the vascular or neurodegenerative features are more associated with cognitive impairments requires further testing. CT imaging features of brain frailty and SVD predict prognosis, should be considered as components of minimization in clinical trials, and may aid clinical decision-making in the future.

## Glossary

CI: confidence interval
ENOS: Efficacy of Nitric Oxide in Stroke
GTN: glyceryl trinitrate
IST-3: Third International Stroke Trial
LACI-2: Lacunar Intervention Trial-2
LACS: lacunar syndrome
MD: mean difference
mRS: modified Rankin Scale
OCSP: Oxfordshire Community Stroke Project
OR: odds ratio
RIGHT-2: Rapid Intervention With Glyceryl Trinitrate in Hypertensive Stroke Trial–2
SPS3: Secondary Prevention of Small Subcortical Strokes
SSS: Scandinavian Stroke Scale
STIR: Stroke Imaging Repository
SVD: small vessel disease
t-MMSE: telephone Mini-Mental State Examination
TICS-M: modified Telephone Interview for Cognitive Status
VISTA: Virtual International Stroke Trials Archive
